# Trophectoderm Biopsy: Present State of the Art

**DOI:** 10.3390/genes16020134

**Published:** 2025-01-24

**Authors:** Anick De Vos, Neelke De Munck

**Affiliations:** 1 Brussels IVF, Universitair Ziekenhuis Brussel (UZ Brussel), Laarbeeklaan 101, 1090 Brussels, Belgium; neelke.demunck@uzbrussel.be; 2 Research Group Genetics, Reproduction and Development (GRAD), Vrije Universiteit Brussel (VUB), Laarbeeklaan 101, 1090 Brussels, Belgium

**Keywords:** trophectoderm biopsy, blastocyst, preimplantation genetic testing, micromanipulation

## Abstract

Trophectoderm (TE) biopsy is at present the most widely used procedure for preimplantation genetic testing (PGT). At the blastocyst stage, more TE cells (five to seven) can be obtained for genetic analysis. While removing TE cells and not touching the inner cell mass (ICM), the procedure is less invasive. Due to a natural selection happening between day 3 and day 5, 6 or 7 of human embryo development, fewer embryos will have to be biopsied and tested. An additional benefit, especially in view of aneuploidy testing (PGT-A), is the lower level of mosaicism present at the blastocyst stage. The biopsy procedure involves two steps: laser-assisted zona pellucida (ZP) opening and the excision of five to eight TE cells from the blastocyst with or without additional laser energy. Different protocols have emerged over time with variations regarding the technique, the exact moment of ZP opening, and the method of cell removal. The ‘pulling’ method involves laser excision, whereas the ‘flicking’ method represents a mechanical approach with or without laser assistance. Embryo developmental speed reaching the full/expanded or hatching/hatched blastocyst stage dictates the timing of the procedure, mostly on day 5 post-insemination, and to a lesser extent on day 6 or even on day 7. The inclusion of lesser quality or delayed blastocysts may impact the quality of the TE sample as well as the clinical outcome. Intracytoplasmic sperm injection (ICSI) is still the preferred method of fertilization for PGT-M (monogenic disorders) and PGT-SR (structural rearrangements). However, conventional in vitro fertilization (IVF) seems feasible for PGT-A (aneuploidy testing). In the absence of a (conclusive) genetic result, the re-biopsy of cryopreserved blastocysts is possible, however, with reduced clinical outcomes. So far, neonatal outcome post-TE biopsy has so far been reassuringly documented.

## 1. Introduction

**Figure 1:**
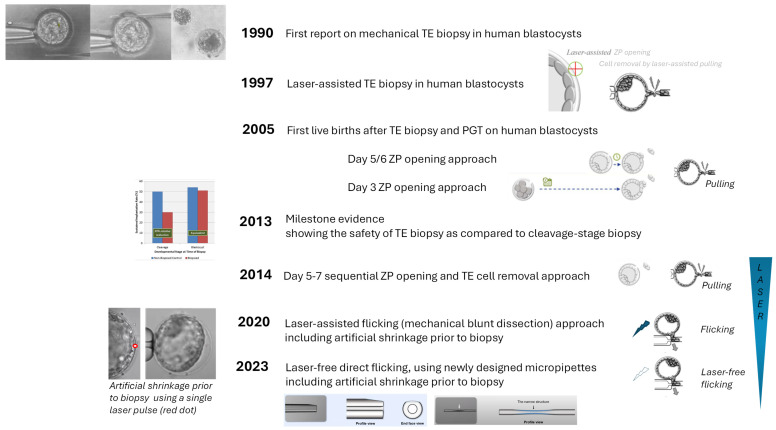
Illustrated timeline on the current state of the art protocols for TE biopsy.

The present review intends to touch upon all the different aspects of TE biopsy: different protocols of the procedure, timing of TE biopsy, cellularity and quality of the sample, inclusion criteria for TE biopsy, concordance between TE and ICM, the preferred or recommended fertilization method to be used, re-biopsy and clinical and neonatal outcomes post-TE biopsy. The review is based on recent peer-reviewed literature and includes personal in-house experience wherever available. A detailed history of the procedure up to 2020 has expertly been outlined recently [1]. Here, we add more recent changes in the procedure, including the available scientific evidence to prove their safety and/or efficacy. An illustrated timeline is presented in Figure 1.

TE biopsy in human blastocysts was first described by Dokras and colleagues in 1990 [2], showing the feasibility of the procedure and that sufficient extra-embryonic material can be obtained for PGT without impairing further development of the blastocyst. At that time, a mechanical zona pellucida opening was used, making a slit opposite of the ICM, so that exclusively TE cells would herniate (i.e., expelling from the zona pellucida). The larger the opening, the more cells herniated about 18–24 h later. The removal of some TE cells was performed mechanically, when the size of the herniation was approximately the same as the blastocyst, using a siliconized glass needle bent at two right angles. The end of the needle was gently rubbed across the waist of the herniation against the bottom of the dish. The rather important notion that the removal of too many TE cells could possibly impair embryo implantation potential was already raised by the authors at that time. The introduction of a laser for micromanipulation techniques on human oocytes (to improve fertilization) and embryos (to help the hatching process and thus improve implantation) in 1995 [3] has changed the biopsy procedure. Laser blastocyst biopsy for PGT in the human, including both laser-assisted zona opening and the laser-assisted excision of some TE cells, was first described in 1997 [4]. Zona pellucida (ZP) opening was performed on day 5/6 on fully expanded blastocysts, entailing further culture and thus some waiting time until herniation. When performing laser-assisted excision of the TE cells, care was taken to target the laser shots to the junctions between the cells that are stretched by pulling the TE in the aspiration pipette. When the first pregnancies and live births after TE biopsy and the PGT of human blastocysts were described [5,6], laser zona opening was the method of choice, being less aggressive, easier and faster than acidic Tyrode or mechanical zona opening. Kokkali et al. [5] stuck to the day 5/6 ZP opening, in contrast, McArthur et al. [6] first described a day 3 ZP opening approach. Back in 2005, the two options for TE cell removal—pulling or flicking—were already described in detail [6], the latter being more appropriate for fully hatched blastocysts.

More recently, Capalbo et al. [7] introduced a ‘day 5–7 sequential ZP opening and TE cells removal’ approach. Simultaneous laser ZP drilling is followed by immediate TE cell removal by means of laser-assisted pulling. As such, the embryo is left undisturbed throughout its preimplantation development. As compared to day 3 ZP opening, ICM herniation and complete hatching can be avoided. Also, it avoids time investment in hatching cleavage-stage embryos that finally do not reach a sufficient quality blastocyst for biopsy. Simultaneous ZP drilling and TE biopsy has also been described in combination with a mechanical blunt dissection approach for cell removal [8]. This flicking method may reduce thermal damage to the TE because fewer laser pulses are used compared with the traditional laser-assisted pulling biopsy technique. Whereas the flicking method used to be described in combination with some or limited lasering, most recently, a novel TE biopsy method independent of laser pulses (at least for cell removal) has been introduced [9]. Indeed, the use of multiple laser pulses at high laser energy levels may cause thermal damage not only to the remaining blastocyst but also to the biopsy sample, possibly introducing DNA damage. A direct flicking method would fully eliminate this concern. The method has been demonstrated in a stepwise manner for fully expanded, hatching and hatched blastocysts and makes use of innovatively designed micropipettes. An inclined plane on the outer wall surface of the holding pipette ensures stability when the biopsy pipette contacts the holding pipette, thus preventing slipping during TE cell detachment. A narrow structure inside the biopsy pipette is designed to trap released TE cells and thus to prevent sample loss.

Ten years of PGT Consortium data collection [10] showed only a minority of TE biopsies (0.5%) between 2003 and 2010 in relation to other biopsy techniques (including polar body and cleavage-stage biopsy). From 2010 onwards, TE biopsy gradually replaced cleavage-stage biopsy in the first place to overcome the high frequency of mosaicism at the cleavage stage, making this earlier stage less appropriate for PGT-A. At least equally important, the well-known invasiveness of two-cell biopsy (more than one-cell biopsy) at the cleavage stage [11,12] could be avoided. In a randomized and paired clinical trial, Scott et al. [13] provided milestone evidence that one-cell cleavage-stage biopsy impairs human embryonic implantation potential (39% relative reduction), while blastocyst biopsy does not. Therefore, the latter is the preferred approach for PGT embryonic sampling. As evidenced by the latest PGT Consortium data collection [14], the application of TE biopsy is gradually rising for PGT-M (from 19% in 2016–2017 to 33% in 2018), is status quo for PGT-SR (30–33%), and has become the most used biopsy stage for PGT-A (98%) and for concurrent PGT-M/SR with PGT-A (96%).

**Figure 2:**
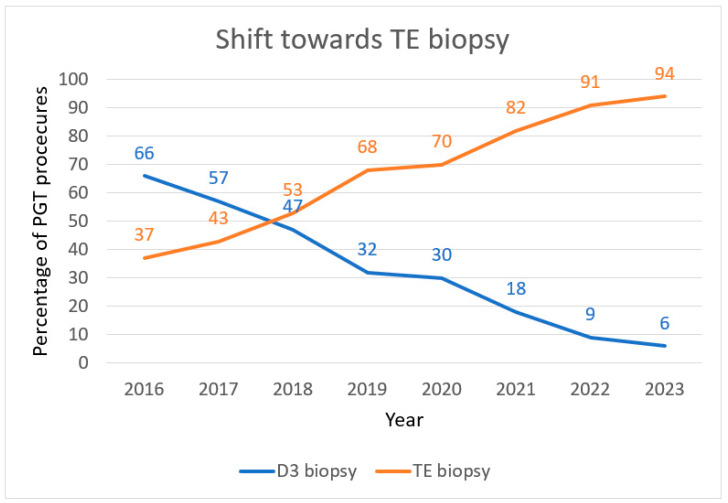
Gradual shift between day 3 cleavage-stage biopsy and TE biopsy from 2016 up to 2023 at Brussels IVF. TE biopsy was started in 2014 in our center. In 2016, it represented the sampling method in 37% of our PGT procedures. This gradually went up to 94% in 2023.

After a long experience with day 3 biopsy, we started TE biopsy at Brussels IVF in 2014. Our approach involved day 4 laser ZP opening and day 5/6 TE cell retrieval by laser-assisted pulling. In 2014 and 2015, about 3.7 blastocysts per biopsy cycle were biopsied, two thirds on day 5 and one third on day 6. Day 7 blastocysts were only included more recently (August 2023). Depending on the PGT indication, about 49% of the tested blastocysts were genetically transferable, resulting in a clinical pregnancy rate of 40.2% post vitrified-warmed blastocyst transfer (two-year experience 2014–2015). Figure 2 illustrates the gradual shift between day 3 and TE biopsy from 2016 up to 2023. The clinical outcome of our PGT activity prior to and beyond this shift was recently summarized [15]. On average, 40–50% of fertilized oocytes can be biopsied on day 5/6. About 14% of cycles scheduled for TE biopsy are cancelled, as embryos do not reach the blastocyst stage or embryo quality is insufficient for biopsy. The biopsy cancellation rate is obviously related to maternal age, retrieved oocyte number and infertility indication. Clinical pregnancy rates reached 50% per frozen blastocyst transfer (2016–2018) [15].

## 2. TE Biopsy Procedure

### 2.1. Zona Pellucida Opening

Laser-assisted ZP opening for TE biopsy was first described in 1997 [4], as efficacy and safety using a 1.48-microns diode laser beam for ZP microdissection was reported shortly before [3]. As it is the most user-friendly (as compared to mechanical ZP opening) and less aggressive than using acidic Tyrode’s solution for chemical ZP opening [16], laser-assisted ZP opening represents the most widely used approach prior to TE cell removal. Upon ZP opening, as the blastocyst and trophectoderm grow, the trophectoderm will start to herniate through the opening.

When ZP opening is performed on day 3 or day 4 of embryo development, herniating blastocysts can be biopsied on day 5 or on day 6 [6]. Although the location of the ICM cannot be predicted, ICM herniation interfering with TE cell removal is limited. In our experience, the risk of ICM herniation does not exceed 10% of all day 4 artificially hatched blastocysts. Much in contrast to day 3 cleavage-stage biopsy, creating a second hole on the opposite side is feasible to proceed with TE biopsy in these cases. Needless to say, this additional manipulation should be limited to a minimum. Allowing extra time for additional blastocyst growth and herniation may result in a reachable TE area, away from the ICM, presenting with a sufficient cell number for sampling.

Reported day 3 openings may vary from 5 µm [17], 6–9 µm [18] up to 25–30 µm (comparable to cleavage-stage biopsy) [6]. The notion holds that the larger the opening the more TE cells herniate, resulting in fully hatched or peanut-shaped blastocysts rather than eight-shaped blastocysts. At Brussels IVF, we aim for +/- 10 µm ZP openings to obtain 8-shaped blastocysts, allowing an easier stretch of the TE when pulling is the method of choice for cell removal. Occasionally, fully hatched (zona-free) blastocysts are obtained. These are then biopsied using the flicking method. With our approach of ZP opening, we never encounter peanut-shaped blastocysts.

When postponing ZP opening until day 5 or day 6 at the blastocyst stage [5], the ICM is visible and can be held on the opposite side. However, time is needed for herniation (1 to 4 h), which may not fit into a busy IVF clinic with plenty of micromanipulation (ICSI and biopsy) procedures. Expanding blastocysts may present with limited blastocoel cavity tension requiring time for sufficient herniation. On the other hand, expanded blastocysts presenting with extremely thinned ZPs may suddenly fully collapse upon ZP drilling even with limited laser energy. Whenever distinction between TE and ICM is no longer possible, re-expansion is needed, including additional waiting time. Day 5/6 ZP openings are created using the lowest laser energy setting in order not to damage the TE and to avoid sudden blastocyst collapse.

As an alternative, it can be opted not to hatch embryos and leave them undisturbed throughout their preimplantation development in vitro until sufficient TE cell numbers and expansion are reached [7]. Then, ZP opening at the opposite side of the ICM is immediately followed by TE cell removal by laser-assisted pulling. This approach is known as ‘day 5–7 sequential ZP opening and TE cells retrieval’. A 10–20 µm opening in the ZP, with the ICM at the 7 o’clock position, is made with a series of laser pulses, working inwards from the outer surface of the zona. Care is taken not to damage the blastocyst. Gently expelling medium through the breach releases the TE cells from the internal surface of the zona, helping to avoid blastocyst collapse during subsequent TE cell removal by pulling. Both Yang et al. [8] and Xue et al. [9] describe ‘artificial shrinkage’ of the blastocyst, using a single laser pulse targeted in between two TE cells, prior to sequential ZP opening and TE cell removal by means of the flicking method. Detaching the TE from the inner part of the ZP by blowing some media through the opening (also known as ‘perivitelline space dilatation’) is only needed for non-collapsed blastocysts.

ZP opening at a later stage in embryo development not only avoids ICM herniation and complete hatching, it may also save the time otherwise invested in hatching cleavage-stage embryos that finally do not reach a sufficient quality blastocyst for biopsy. To what extent the day 3 or day 4 created hole in the ZP impairs the blastocyst growth, expansion and hatching process afterwards, between cleavage and blastocyst stage, is scarcely documented. One randomized control trial (RCT) [19] and one retrospective single-center cohort study [20] reported significantly more blastocysts biopsied when hatching was only performed on day 5/6. This finding was not confirmed by Yang et al. [21], showing similar percentages of biopsied blastocysts per fertilized oocyte regardless of the moment of ZP opening. On the contrary, one other retrospective cohort study reported a significantly higher mean number of biopsied blastocysts in the day 3 ZP opening group [22], however, with potential bias in the significantly higher number of retrieved oocytes in that group. Zhao et al. [19] showed that the mosaic blastocyst rate was not influenced by the moment of ZP opening. In contrast, significantly increased mosaicism after D3 ZP opening (19.6% versus 8.1%) was reported by Xiong et al. [22]; however, a potential bias lies in the different TE cell removal strategies used in the two study groups (laser-assisted flicking for day 3 ZP opening and laser-free flicking for day 5/6 ZP opening). In contrast, Yang et al. [21] reported a lower rate of mosaic blastocysts with day 3 ZP opening. So far, a clear explanation for this controversy is missing.

Limited evidence exists so far to show better clinical results with day 5/6 ZP opening, which was reviewed and meta-analyzed by Cimadomo et al. [23]. The concluded higher live birth rates per euploid single embryo transfer for day 5/6 ZP opening are fully on behalf of one large retrospective single-center cohort study [20], where infertility diagnosis still represents a major possible confounder. Additional bias might have been introduced by the ‘one-fourth ZP ablation’ only performed in the sequential TE biopsy study arm. The rationale is to avoid embryo trapping (and thus monozygotic twinning), leaving sufficient ZP protection to the embryo. Additional evidence for a better clinical outcome with delayed ZP opening as reported by Yang et al. [21] might be related to different embryo qualities in both study groups. There is a clear need for more and larger RCTs than the one by Zhao et al. [19] for firm conclusions on this matter. Importantly, similar miscarriage rates per clinical pregnancy, irrespective of the moment of ZP opening, were reported in all three studies [19,20,22] within the meta-analysis [23] and by Yang et al. [21].

### 2.2. TE Cell Removal

Two options exist for TE cell removal: the ‘pulling’ and the ‘flicking’ method [6], as illustrated in Cimadomo et al. [1]. Pulling has been the standard for a long time with occasional flicking depending on the rigidity of the trophectoderm or the hatching stage of the blastocyst (far or fully hatched).

The pulling option is ideal for partially hatched (figure eight-shaped) blastocysts. Herniating TE cells are aspirated and stretched to form a narrow bridge of TE cells between the aspirated cells and the cells still within the ZP. A few laser shots are fired onto the narrow bridge of cells, close to the biopsy pipette, taking care to specifically target the cell junctions instead of firing in the middle of a cell. It is preferred to fire individual pulses so that each time the operator can anticipate on how the blastocyst reacts. The number of pulses needed is difficult to specify and case-dependent (on average three to five pulses or sometimes more). Once detached, the biopsy sample can be removed carefully by pulling alone, as such limiting the number of pulses. Care is taken to minimally damage the TE of the blastocyst or the cells of the biopsy sample.

Occasionally, after multiple laser shots, the TE can become extremely rigid, rendering all additional pulses inefficient and without further impact. To minimize excessive lasering and excessive manipulation, in these cases, one can switch to mechanical biopsy or the ‘flicking’ method, by rubbing the created TE bridge against the holding pipette. Using a vigorous downwards movement, the aspirated TE cells are detached. The latter method is also appropriate for half or more hatching blastocysts to avoid pulling the blastocyst completely out of its protective ZP. Fully hatched, zona-free blastocysts should be biopsied with the mechanical ‘flicking’ technique. Indeed, these blastocysts represent with a huge volume. Limited pulling would not allow for creating a sufficiently narrow waist of TE cells to allow excision with a limited number of laser pulses. Therefore, to minimize manipulation and limit laser firing, only the necessary number of TE cells is aspirated, and a few laser pulses close to the aspiration pipette suffice to loosen the cell bonds before starting the rubbing.

Normally, flicking involves some laser pulses to loosen cell bonds between the selected TE cells and the rest of the blastocyst [8]. However, most recently, a TE flicking method without laser pulses was introduced [9], as such, fully avoiding thermal damage to the TE cells of both the blastocyst and sample. An innovatively designed holding pipette (including an inclined plane on the outer wall surface of its opening end) to increase stability during mechanical friction was used. The aspiration pipette was designed to prevent sample loss (reporting a reduction from 18%, at least for trainees, to 0%). The former figure needs to be framed in that with experienced operators in our laboratory, sample loss during TE biopsy is less than 2%, even without the use of specially designed aspiration pipettes. Laser-free flicking should be considered with caution, as it represents a less gentle and less precise sampling technique. Its impact on blastocyst integrity and biopsy sample quality needs to be validated both compared to the pulling method and compared to the flicking method complemented by a few laser pulses to ease the biopsy and to precisely control the target number of TE cells to be detached.

At present, one single prospective observational study has compared the pulling and laser-assisted flicking strategies in terms of artefactual mosaicism [24], using next-generation sequencing (NGS) as a diagnostic approach. Both approaches were compared in combination with day 3 ZP opening. The authors concluded that no specific method—pulling or laser-assisted flicking—would increase the generation of artefactual mosaicism. The bottom line is that biopsy and tubing procedures need to be performed following standardized high-quality procedures. Regardless of the TE cell removal methodology, the aim should always be minimizing blastocyst manipulation. No association with clinical pregnancy was observed for the number of laser pulses used nor for the technique used (pulling or flicking) [24].

When retrospectively comparing laser-assisted (3–5 pulses) TE cell removal by flicking with a quick flicking movement (usually performed without laser-assistance, as stated by the authors), Xiong et al. [22] reported a significantly higher mosaic blastocyst rate with the former approach (19.6% versus 8.1%). However, potential bias lies in the different moment of ZP opening between both study groups (respectively day 3 or day 5/6).

**Figure 3:**
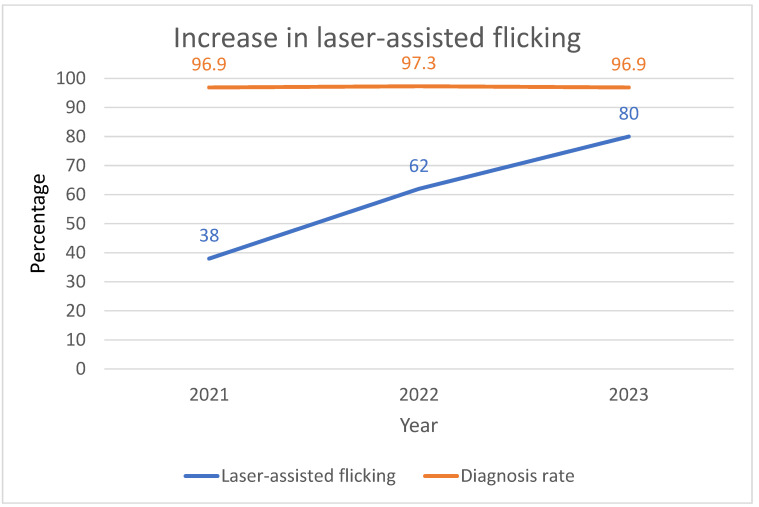
Increase in laser-assisted flicking (percentage of all PGT procedures) since 2021 at Brussels IVF. The diagnosis rate (percentage of samples with a conclusive diagnosis) was independent of the increasing proportion of laser-assisted flicking.

At Brussels IVF, pulling used to be the standard method for TE cell removal with occasional laser-assisted flicking in cases of rigid TE or for fully hatched blastocysts. However, since we started to register the biopsy method in November 2021, the proportion of flicking (always complemented with a few laser pulses) only increased: it was 38% in 2021, 62% in 2022 and 80% in 2023 (Figure 3). Whereas the hatching stage of the blastocyst and the rigidity of the TE may determine the method of choice, operators may find the technique that works best for them and suits the embryo. Among twelve operators, four of them conservatively stick to the pulling method in a high percentage of blastocysts biopsied (only 15–42% of biopsied blastocysts involve laser-assisted flicking), whereas six operators use flicking for 70% or more of the blastocysts biopsied. Two operators use both methods in an equal proportion. In our experience, the diagnosis rate per blastocyst biopsied (>=95% overall) is operator independent (as evaluated on a yearly basis) but also method independent, as illustrated in Figure 3.

### 2.3. Biopsy Sample: Cell Number and Quality

The number of TE cells removed during a biopsy should serve two requisites that need to be balanced: the sample should allow good-quality molecular diagnosis to be conclusive; however, on the other hand, the putative impact on embryo competence and viability should be minimal. TE biopsy entails the collection of a biopsy sample of 5 to 10 cells [25]. Reportedly, seven to eight cells in the sample are considered reasonable to achieve good molecular analyses [26,27,28]. The definition of a threshold number of TE cells to be retrieved without clinical impact is based on three studies, investigating the impact of the removed cell number on implantation rates [27,29,30].

Zhang et al. [27] illustrated that specifically in blastocysts with poor TE morphological score (grades B and C), the implantation potential was negatively affected by the biopsied TE cell number. The importance of obtaining appropriately sized TE biopsies was further underlined by Neal et al. [29], showing that TE biopsies with the highest relative DNA content were associated with lower live birth rates after single embryo transfer. The study used relative DNA content as a surrogate proxy for biopsy size, acknowledging that the method was unable to precisely quantify the number of cells removed at the time of TE biopsy. Additional evidence from a retrospective cohort study showed that when a large amount of TE cells was biopsied (on average 10 cells), this negatively impacted on implantation rates [30]. It seems important not only to define the ideal blastocyst expansion stage but also to critically evaluate the actual overall number of TE cells available in the TE prior to biopsy. Removing seven to eight cells from a grade A TE (on average 80 to 100 cells) obviously represents a lower embryo biomass reduction as compared to when a grade B TE (on average 40 to 50 cells) is biopsied.

It needs to be acknowledged that the cell number in the sample does not always correlate to its size: large biopsy pieces may contain few cells, depending on the cellularity of the whole TE. Premature biopsy timing on less expanded blastocysts, with less and bigger cells constituting the whole TE, is therefore not recommended. On the other hand, when biopsying blastocysts with high cell numbers in the whole TE, the removal of smaller biopsy pieces may suffice to obtain the required cell number for analysis. Biopsy operators should precisely and effectively control the target number of TE cells to be retrieved. At Brussels IVF, within a research setting, the precision of cell number retrieved was recently compared between three biopsy procedures (pulling, laser-assisted flicking and direct flicking). It was observed that no difference exists between the two former methods (8.0 +/- 3.7 and 7.5 +/- 2.2 cells removed, respectively) (unpublished data), given that the flicking method involves some laser-assisted cell bound loosening prior to detachment. Laser-free flicking resulted in significantly more cells biopsied (9.7 +/- 4.1). These results show that direct flicking is less precise in determining the cell number that will be detached. In this respect, it seems also important to validate its operational impact on fair- and poor-quality blastocysts, which has not been documented so far [31,32].

Apart from the biopsy sample cellularity, the morphological quality of the biopsy specimen may impact the quality of the genetic test outcome, as recently illustrated [33]. The morphology of biopsied TE cells was correlated to their quality and subsequent testing result, showing that rather than cell number input, cell quality is one of the primary factors associated with the quality of the genetic test outcome. High- or good-quality TE cells generate excellent data uniformity and lower allele drop out (ADO) rates, whereas low-quality TE cells are responsible for ‘background noise’. Usually, biopsy specimens are directly used for downstream genetic analysis without other processing. Whether intermediate processing by morphology based on cell sorting prior to genetic analysis (reducing technical noise through ‘ideal’ sample collection and low-quality sample elimination) is a desirable approach to take remains to be seen if not quite questionable in terms of representation sample/remaining blastocyst.

When comparing the three biopsy methods, similar percentages of apoptosis were observed in the biopsy samples, whereas the direct flicking method resulted in a significantly higher percentage of necrotic cells in the sample (unpublished data). The latter finding did not impact successful whole genome amplification (WGA) and subsequent chromosome analysis. However, since procedure-induced necrosis in the remaining blastocyst should be avoided, we have not adopted the direct flicking method within our clinical setting.

To summarize the current state of the TE biopsy procedure, laser-assisted ZP opening represents the standard. Both approaches, early day 3 or day 4 opening and day 5–7 sequential ZP opening followed by immediate cell removal, are in use with so far insufficient randomized evidence favoring one or the other. Preferentially, TE cell removal is achieved by laser-assisted pulling or laser-assisted mechanical flicking, always aiming at minimizing blastocyst manipulation and excessive laser application to avoid thermal damage. Laser-free flicking represents a less gentle and less precise sampling technique. The biopsy sample (in cell number and quality) should serve a minimal diagnostic failure rate without impairing the implantation potential of the remaining blastocyst.

## 3. Inclusion Criteria for TE Biopsy

### 3.1. Poor-Quality Blastocysts

In our laboratory at Brussels IVF, using sequential culture media, a blastocyst formation rate of about 45–50% per fertilized oocyte is obtained (data from 2015). Of these, 10–15% are excellent quality (at least Gardner stage 3, ICM grade A or B, TE grade A [34]) and the remaining 30% are good quality (at least Gardner stage 3, ICM grade A or B, TE grade B [34]) or early blastocysts (BL1, BL2). These PGT patients were on average 33 years of age and presented with an average of 12 cumulus–oocyte complexes. These figures are in line with documented laboratory key performance indicators (KPIs) [35]. Obviously, maternal age and poor prognosis infertility impact these figures. Within a PGT population, the presence of an underlying infertility indication may impact the blastocyst formation rate and thus the biopsy cancellation rate (which in our experience ranges from 10 to 18%). Patients with cancelled biopsy cycles are on average 2 years older and have on average fewer oocytes available to start from compared to patients with at least one blastocyst available for biopsy.

We used to be quite stringent on the inclusion of blastocysts for TE biopsy after assisted hatching on day 4: only expanding/hatching and expanded/hatched blastocysts with ICM grade A or B and TE grade A or B were included for biopsy on day 5. Occasionally, by exception, grade CA blastocysts could be included for biopsy. Cavitating morulae and early or full blastocysts remained in culture and biopsy was attempted 24 h later, on day 6 of development, based on the same criteria used on day 5. Poor-quality blastocysts (grade C for either ICM or TE) and delayed day 7 blastocysts were never included until recently (2023 onwards). In respect to blastocyst quality, this involves the inclusion of grade CB and eventually grade AC and BC blastocysts for TE biopsy.

It needs to be acknowledged that poor-quality blastocysts have lower euploidy rates: 37.5% [36]; 25.5% [7]; 23.5% [37] and also reported by Minasi et al. [38]. However, when these euploid blastocysts were transferred, no significant difference was found in the implantation rate in relation to morphology: 49.1%, 59.4%, 43.3%, and 53.8% for excellent-, good-, average- and poor-quality blastocysts, respectively [7]. When clinically used, an overall 2.6% increase in the number of live births can be expected (and even a 5% increase in women aged over 42 years) [37]. Therefore, this modest clinical benefit to live birth should not be underestimated nor neglected, specifically not in older patients with fewer or no good-quality blastocysts available. However, patients should be aware of the reduced prognosis, including an increased miscarriage rate (36.4% versus 13.9% with good-quality blastocysts) [37]. If poor-quality blastocysts are to be included in clinical practice, PGT-A is recommended by the authors.

At Brussels IVF, we have too limited experience so far with the inclusion of poor-quality blastocysts within our PGT practice to validly add to these data.

### 3.2. Delayed Day 7 Blastocysts

The rate of human embryo development in vitro is well known to be asynchronous, not only in morphological quality but also in degrees of expansion, which impacts the timing of TE biopsy. The timing of blastocyst expansion can vary by over 24 h and occur on day 5 or on day 6 (respectively, 65 and 35% of our biopsied blastocyst population) or even on day 7. Day 7 blastocysts make up only 5% of useable blastocysts [39] (based on seven publications): a small, but clinically significant group of embryos.

In respect to PGT-A, only a small proportion of blastocysts reach a suitable stage for biopsy on day 7 (2–8%). Euploidy rates between 25 and 49% have been reported (according to their morphology—fair or excellent), averaging 34%, based on four publications reviewed in [39]. These data have been confirmed: 43% euploid day 7 blastocysts [40] and 24.4% euploid blastocysts 156–168 h post-insemination [41].

Day 7 (euploid) blastocysts have reported live birth rates between 11 and 42% [42,43,44] after frozen-thawed embryo transfer. Sustained implantation rate after euploid day 7 single embryo transfer appeared slightly lower compared to day 5 (52.6% versus 68.9%) or day 6 (66.8%); however, these were not significantly different (*p* = 0.14) [40]. The clinical value of day 7 euploid blastocysts was further underscored by Cimadomo et al. [41], showing a 7.3% relative reduction in the number of patients obtaining euploid blastocysts and a 4.4% relative reduction in the number of patients obtaining live births if embryo culture would end on day 6.

It should however not be neglected that increased pregnancy loss with day 7 euploid blastocysts has been reported: 11.5–14.9–19.2% for day 5/6/7 blastocysts, respectively [45]; 22–40% beyond 144 h post-insemination [41].

Limited numbers of live births after day 7 blastocyst frozen embryo transfers have been reported so far (*n* = 58 in [39]; *n* = 25 in [45]; *n* = 10 in [41]). Reported similar perinatal outcomes compared to day 5 and day 6 blastocysts are limited to eight live births [42].

At Brussels IVF, we have only biopsied 79 day 7 blastocysts (i.e., only 3% of all blastocysts biopsied as compared to 15% in [41]) in 64 PGT cycles (8% of all PGT cycles in 2023). The rate of genetically transferable embryos was 45%. So far, we only transferred 11 of these embryos, resulting in a low clinical pregnancy rate (18.2%). The miscarriage rate was equally high (18.2%), confirming the above data, albeit in extremely low numbers.

To summarize Section 3, not all blastocysts blastulate at the same rate and quality. Low-quality blastocysts and delayed day 7 blastocysts are however not all incompetent, resulting in healthy infants. Opting them in to increase the pool of transferable embryos should include proper counselling about their reduced prognosis. Additionally, limited information exists on obstetric and perinatal outcomes, and on the long-term health implications for the offspring, as has been raised in a recent ‘fertile battle’ on this topic [46].

## 4. Concordance Between TE and ICM

The chromosomal concordance between TE and ICM has been reported to be as high as 96.7% (based on four studies reviewed in [47]). Using next-generation sequencing (NGS), featuring higher sensitivity and precision, these data have been confirmed, at least for whole chromosome aneuploidies (96.8% concordant, 90/93), which are believed to be in the majority derived from meiotic errors in the oocyte [48]. Likewise for maternal or paternal inherited monogenic disorders or structural rearrangements (PGT-M and PGT-SR), a similar quasi-full concordance between TE and ICM for the tested condition can be expected.

In contrast to whole chromosome aneuploidies, Victor et al. [48] reported a significant drop in concordance for segmental aneuploidies (only 42.9%, *n* = 7), which was most probably due to their mitotic origin. Mitotic errors during the first cleavage divisions result in mosaicism within the preimplantation embryo, ultimately containing cell lines with different karyotypes [47]. The earlier the error occurs in preimplantation development, the more cells in the blastocyst will be affected with the error. If an error occurs later in preimplantation development, fewer blastocyst cells will carry the error. As such, high-grade and low-grade mosaicism can be anticipated [47,49].

The diagnosis of mosaicism has become challenging due to both technical and biological issues. The predictive utility of PGT-A implies the premise that the ploidy status of the TE biopsy faithfully represents the entirety of the blastocyst [50] but more specifically of the ICM. Whereas it is sufficient to diagnose meiotic aneuploidies, mathematical modeling has shown that five TE cells cannot inform on the status of a complete embryo [51,52]. This is related to the fact that a large majority of aneuploidies at the blastocyst stage are of mitotic origin; they are both clonal and insular. The concordance found between TE and ICM in cases of TE biopsies displaying mosaicism is indeed lower than expected and related to the type (whole chromosome or segmental) and the level of mosaicism (reviewed in [53]). Given that mosaic diagnoses frequently result in healthy live births, different ‘self-correction mechanisms’ have been proposed to explain how conceptuses become more chromosomally normal as development progresses [49]. Among these are the preferential apoptosis of aneuploid cells as evidenced by apoptotic remnants present in the blastocoel fluid [54]. The enrichment of aneuploid cells to the TE compartment as compared to the ICM was not reported by Starostik et al. [55]; however, most recently, Griffin et al. [56] showed that aneuploid cells were sequestered away from the ICM and partly to the TE. The authors calculated a positive predictive value of 86.7% (95%CI 77.8–92.4%) for the ploidy of the ICM, classifying the TE result as a ‘relative accurate predictor’ of the ICM karyotype.

To a higher extent, aneuploid cells were more significantly sequestered to the blastocoel fluid within the blastocoel cavity and to the peripheral cells not participating in blastocyst formation [56]. Indeed, partial compaction as part of ‘self-correction’ to exclude aneuploid cells away from the embryo proper has been reported [57]. The analysis of the excluded cells from euploid blastocysts showed that these were often aneuploid or with highly fragmented DNA. In respect to TE biopsy, it seems important to avoid the inclusion of peripheral cells (that are squeezed between the TE and the ZP) and/or blastocoelic apoptotic remnants into the sample, specifically for PGT-A and when biopsying poor-quality blastocysts.

Since chromosomal mosaicism is a common feature of early human development, the transfer of mosaic embryos, particularly in the absence of euploid embryos after PGT-A, is a growing practice in IVF. Different scientific societies have published guidelines or position statements [58,59] and reviewed in [60], including prioritization criteria and recommendations regarding prenatal diagnosis in these cases. It needs to be acknowledged that the transfer of mosaic embryos may lead to fetal mosaicism and/or placental mosaicism. Fetal mosaicism is detectable by ultrasound when abnormalities are present or with invasive amniocentesis, being the most representative of the chromosomal complement of the fetus. In contrast, non-invasive prenatal testing (NIPT) or chorion villus sampling (CVS) may only reveal the placental chromosome constitution, which could differ from the actual fetal chromosome set. When confined placental mosaicism (CPM, mosaicism solely located within the placenta) is detected, the importance of more rigorous antenatal monitoring needs to be highlighted, as a higher risk of compromised fetal growth has been associated with CPM [61,62].

## 5. Fertilization Method and Abnormal Ploidy

As stated in the ESHRE PGT Consortium and SIG Embryology good practice recommendation for embryo biopsy for PGT (2020) [25], intracytoplasmic sperm injection (ICSI) is preferable for PGT to minimize the risk of both maternal contamination from residual cumulus cells and paternal contamination from surplus sperm cells attached to the ZP. The careful removal of cumulus cells (denudation) and rinsing of oocytes prior to ICSI (or of zygotes in case of conventional IVF prior to fertilization check), are critical to avoid residual maternal contamination in the biopsy sample. Additional to eliminating potential parental contamination, the use of ICSI (in contrast to conventional IVF) also ensures monospermic fertilization. The committee opinion of the American Society of Reproductive Medicine (ASRM) on the use of ICSI for non-male indications published in the same year [63] states it slightly different in that ICSI for PGT in the absence of male factor infertility should be limited to cases where the contamination of extraneous sperm could affect the accuracy of the test results.

Before promoting the use of conventional IVF for PGT, some fundamental issues had to be clarified with respect to PGT-A. In an initial retrospective, small sample-sized study, Palmerola et al. [64] identified a non-significant trend toward a higher rate of mosaicism in IVF (25.9%) versus ICSI (20.9%). The underlying mechanism was not well understood at that time. A technical artefact related to genetic contamination, either resulting from maternal somatic cumulus cells or adherent sperm cells to the ZP, could not be ruled out. The unique packaging of sperm DNA may, however, refute the latter source of contamination [65]. Indeed, sperm DNA may fail to amplify under the conditions used for PGT-A on TE biopsy samples, as unequivocally shown by De Munck et al. [66], when using a whole genome amplification (WGA) protocol (PicoPLEX). The authors even demonstrated that sperm cells attached to the ZP remained intact between day 1 and the moment of TE biopsy on day 5. Thus, in the absence of any sign of degeneration, it is concluded that amplification on day 5 of embryo development will be absent. Most recently, Zhang et al. [67] confirmed that sperm DNA failed to amplify under Picoplex and ChromInst conditions (involving milder lysis conditions, which effectively amplify the biopsied TE sample but not the sperm DNA). However, when using multiple displacement amplification (MDA), involving stronger cell lysis conditions, sperm DNA could be amplified (if at least 10 sperm cells were present). At Brussels IVF, in collaboration with our Medical Genetics laboratory, we recently confirmed that the presence of three residual sperm cells affected the MDA amplification signal (unpublished data). When analyzing parental contamination in 150 blastocysts derived from IVF, no paternal contamination was detected in any sample [67]. Maternal contamination was observed in one TE sample at a contamination level of 10% (1/150 = 0.67%). These results are very much in line with Dong et al. [68] showing 0.83% (1/120) maternal contamination and no paternal contamination when 120 IVF derived vitrified blastocysts were analyzed. All together, these results indicate that maternal contamination should be carefully avoided in PGT cycles. Residual cumulus cells should be completely removed before biopsy regardless of whether the oocytes were inseminated by IVF or ICSI (respectively, prior to fertilization check or ICSI). A visual inspection of spermatozoa under an inverted microscope should largely avoid the presence of spermatozoa in biopsied TE samples.

Apart from the parental contamination issue, several sibling oocyte studies have indicated that euploidy rates are similar between IVF and ICSI in the context of non-male or mild male factor infertility [66,69,70] with the latter study showing similar birth rates and miscarriage rates between both insemination procedures. A large retrospective cohort study, however, indicated an 11% lower euploidy rate with ICSI compared to conventional IVF in a non-male factor infertility setting [71]. When adjusting for PGT reference laboratory, data were no longer statistically significant. An even larger retrospective cohort study on non-male factor PGT cycles [72] did not show any difference in the number of embryos suitable for transfer (41.6% and 42.5%, respectively) or in live birth rates (50.1% and 50.8%, respectively) or in pregnancy loss rates (16.6% versus 15.5%). Taken together, these data drive some laboratories to promote the use of conventional IVF, rather than ICSI, as the fertilization method, specifically for PGT-A cycles in the absence of male-factor infertility [65]. This change in approach for PGT-A might be acceptable provided that possible maternal contamination can be controlled for by careful denudation of the oocytes prior to fertilization check, as it is considered to be faster, easier and less invasive as compared to the enzymatic/mechanical removal before ICSI [66].

At Brussels IVF, we still perform ICSI for all PGT cycles, specifically for PGT-M and PGT-SR, but also for PGT-A cycles. This is in line with the latest ESHRE PGT Consortium data collection XXI (PGT analyses in 2018), reporting that ICSI was the main fertilization method (97%) [14]. Whereas paternal contamination resulting from using conventional IVF carries a low risk of an adverse event or misdiagnosis in PGT-A, the risk might be higher with PGT-SR, while PGT-M involves different processing protocols, presenting a different risk profile [73]. Therefore, increased caution and the continued use of ICSI specifically for PGT-SR and PGT-M seems appropriate.

The use of ICSI ensures monospermic fertilization. Within our PGT population, using ICSI as the fertilization method, 2.4% of zygotes display one pronucleus (1 PN) and 1.4% display >=3 PN. A careful fertilization check seems important, as zygotes with two pronuclei would indicate a diploid embryo [74]. However, the limitations of a single static observation need to be recognized [49]: observing 1 PN may be related to asynchronous PN formation or fading or to PN fusion. These phenomena can only be ruled out by time lapse (TL) evaluation, allowing dynamic and continued observation and thus allowing to recognize true 1 PN zygotes [75]. Likewise, observing 0 PN might be related to early pronuclear breakdown, which can also be recognized in TL. However, the PN number is not always an accurate proxy of parental ploidy [76]. Given the fact that 42.98% of 1 PN embryos (in static evaluation) are diploids [76] and that 17% (15/88) of true TL observed 1 PN embryos are diploids [75], their inclusion for TE biopsy needs to be considered, especially when the transferable pool of embryos is limited. The confirmation of biparental inheritance is, however, strongly recommended [75,77], which is not possible with platforms only relying on copy number. It needs to be added, however, that only 2 out of 15 true 1 PN diploid embryos reached a transferable blastocyst stage and only one gave rise to a healthy live birth [76], illustrating the lower prognosis. At Brussels IVF, 3 PN embryos are removed from the biopsy pool, whereas 0 PN and 1 PN embryos might be included for TE biopsy given that the diagnostic test allows to distinguish haploidy (or triploidy) from diploidy (which is the case for SNP array or haplotyping-based approaches).

## 6. Re-Biopsy

Blastocyst biopsy involves a low risk of inconclusive diagnoses, which is reported to be 2–6% for PGT-A cycles [28,78]. However, the diagnostic result rates may differ according to the indication for testing. Whereas similarly high diagnosis rates were reported for PGT-A cycles (98%), PGT-M cycles resulted in a lower diagnostic result rate (87%), as reported by the ESHRE Consortium [14]. However, PGT-M in 2018 still involved 65% cleavage-stage biopsy and mostly PCR as a diagnostic test (85%). A higher diagnostic result rate is obtained when a TE biopsy in combination with SNP array is used for PGT-M (97.2% in 2023, V. Berckmoes, Center for Medical Genetics, UZ Brussel, personal communication).

Test failure may depend on factors such as the day of biopsy (day 5/6/7), stage of blastocyst development, biopsy methodology and operators’ expertise in performing biopsy and tubing [23,28,78]. Despite the low prevalence, test failures do represent a wastage of embryos, and the rescue of these blastocysts needs to be considered. Retesting, however, inevitably involves a second round of biopsy and a second round of vitrification as well [79]. To what extent this practice impacts the developmental potential of blastocysts needs to be carefully analyzed. Blastocysts are capable of withstanding two cycles of vitrification as evidenced by reportedly high re-warm survival rates. Suitability for re-biopsy will depend on their re-expansion and hatching, in part related to their morphological quality and cellularity, more specifically that of the remaining TE. Upon re-biopsy and re-testing, non-negligible percentages of genetically transferable embryos are obtained, ranging from 48 to 66% [28,78,79,80,81]; depending on the genetic indication tested for. These percentages are not different from first-time tested embryos, making their rescue worth the effort.

Several studies have pointed to a reduced chance of pregnancy and/or live birth with double-biopsied blastocysts [79,82,83,84,85]; however, this is non-significant after adjustment or due to small sample sizes in the double-biopsied groups. One larger retrospective study reported a significantly lower live birth rate (LBR) and higher miscarriage rate (MR) after transferring re-biopsied blastocysts compared to those biopsied once [78]. Although an extra round of biopsy and vitrification may cause a detrimental effect on embryo viability, re-analyzing the test-failure blastocysts contributes to increasing the number of blastocysts available for transfer. Their clinical use seems important as healthy live births can result from this practice. Whereas PGT-A patients may opt to proceed with untested embryo transfer, patients with inherited disorders do not have this option and may elect to proceed with the second biopsy and vitrification.

The above-mentioned existing data on clinical outcomes with double-biopsied blastocysts have been meta-analyzed recently [23,86,87]. Based on three included studies, Cimadomo et al. [23] showed a similar LBR per euploid single embryo transfer (SET) and a similar MR per clinical pregnancy in the re-biopsied group (*n* = 86) compared to the single-biopsied group (*n* = 6896). In contrast, enlarging the number of included studies, Bickendorf et al. [86] showed that ‘double biopsy/double vitrification’ (*n* = 385) resulted in reduced live birth rates (seven studies, RR = 0.72, 95% CI 0.63–0.82) compared to ‘single biopsy/single vitrification’ (*n* = 20,579). No significant changes were observed in miscarriage rates. To frame these findings, significant heterogeneity for multiple study outcomes needs to be acknowledged [86]. Potential confounding factors include inclusion criteria for biopsy (including poor-quality and/or delayed blastocysts or not), day of assisted hatching, protocols for both vitrification and biopsy (number of TE cells biopsied, pulling or flicking, overnight culture post-warming before second biopsy) and the skill level of biopsy operators. Significantly reduced live birth rates per embryo transfer after double TE biopsy/double vitrification were confirmed in a similar meta-analysis by our group (OR: 0.51, 95% CI 0.34–0.77, seven studies) [87]. In contrast to Bickendorf et al. [86], we did observe a significantly increased miscarriage rate per clinical pregnancy after the transfer of double-biopsied/double-vitrified blastocysts (OR: 2.08, 95% CI 1.13–3.83, seven studies) [87].

Apart from its clinical value, be it at a lower efficiency, the safety of re-biopsy and re-vitrification should be critically evaluated. Neonatal data following the transfer of double-biopsied blastocysts are extremely scarce and limited to three studies reporting on six [77], nineteen [28] and five newborns [79], respectively. So far, no differences in gestational age or birthweight were observed following re-biopsy/re-vitrification. Obviously, more data need to be gathered to investigate this issue further.

## 7. Obstetric and Neonatal Outcome

Two former meta-analyses available in the literature [88,89] on obstetric and neonatal outcomes post-biopsy suffer from heterogeneity with respect to the stage of biopsy (cleavage or blastocyst). Only a minority of the included studies involved TE biopsy (respectively only 3 out of 15 and 4 out of 19 of the included studies). A recent scoping review, trying to identify gaps and biases, highlighted the additional confounding effect of infertility status and cryopreservation [90]. Many studies have evaluated the effects of embryo or trophectoderm biopsy without distinction between PGT-M (mostly fertile patients), PGT-SR, and PGT-A (used for infertile patients). Cryopreservation has been reported to potentially influence obstetric and neonatal outcomes, and to what extent TE biopsy might add to this impact needs to be established.

In respect to obstetric and neonatal outcomes, Alteri et al. [90] included twelve studies on TE biopsy, which were mostly retrospective cohort studies. A slightly increased likelihood of preterm delivery was reported in one large retrospective cohort study (PGT-M + PGT-A) (adjusted odds ratio 1.20, 95% CI 1.09–1.33) for singletons from the transfer of frozen biopsied embryos (*n* = 6244) as compared to babies derived from non-biopsied blastocysts (*n* = 10,002) [91]. In contrast, seven other studies (for references, see [90]) did not report such a difference (all together involving 2080 and 4138 singletons in the biopsied and non-biopsied group, respectively). Whether significantly reduced serum β-hCG levels on day 12 after the transfer of biopsied blastocysts [92] can be held responsible for abnormalities in placentation, possibly leading to preterm birth, remains controversial, as conflicting data have been reported [93]. No significant difference in the rate of placental anomalies was observed (five studies). A significantly higher risk of gestational hypertension in association with biopsy was observed in one retrospective cohort study (PGT-M + PGT-SR + PGT-A) (adjusted odds ratio 1.94, 95% CI 1.07–3.52) reporting on 241 and 515 singletons in the biopsied and non-biopsied group, respectively [94]. However, statistical significance was only maintained for non-severe forms. In contrast, four other studies (for references, see [90]) did not report this increased risk (all together on 1203 and 2822 singletons in the biopsied and non-biopsied group, respectively). With respect to neonatal outcomes, low and very low birthweight rates (eight studies) and small or large for gestational age rates (two studies) did not differ between biopsied and non-biopsied blastocyst-derived singletons (for references, see [90]).

The best effort in minimizing the above-mentioned heterogeneity was made in a most recent meta-analysis [95], including 13 studies on TE biopsy, including seven in common with [90]. Six studies were performed in the United States, and the other seven were from China. The analysis involved over 11,000 singleton live births after PGT treatment with TE biopsy before embryo transfer. Subgroup analysis was performed on FET cycles (frozen embryo transfer cycles, in contrast to fresh transfers), ICSI (instead of conventional IVF) and single embryo transfers. The confounding effect of infertility status, however, remains, as most studies included have mixed PGT indications. This latest, enlarged meta-analysis [95] confirmed the significantly increased risk of preterm birth (pooled odds ratio 1.12, 95% CI 1.03–1.21). However, no other obstetric outcomes were different between the biopsied and non-biopsied groups. Additionally, no increased risk for low birthweight, small for gestational age nor macrosomia was reported post-TE biopsy. The authors considered the risk for preterm delivery related to placental insufficiency, which may have probably resulted from TE biopsy, as relatively mild. Therefore, in the absence of other increased obstetric and neonatal risks, TE biopsy is considered a safe practice for PGT treatment.

It needs to be added that endometrial preparation for FET cycles may impact obstetric and/or neonatal outcomes post-PGT. When comparing hormone replacement therapy (HRT) FET with natural cycle (NC) FET for PGT, a significantly higher rate of pregnancy-induced hypertension was observed (2.5% versus 0.7%, *p* = 0.022) [96]. As recently summarized, programmed endometrial preparation for FET cycles is associated with an increased risk of large for gestational age and macrosomia compared to natural cycles [97]. As Petch and Crosby [98] stated, some of the risk of pregnancy-induced hypertension following PGT may possibly be mitigated by performing a natural cycle FET, but more study in this area is needed.

Lastly, as the methodology of the biopsy techniques (timing of ZP opening, timing and method of cell removal, be it pulling with laser assistance or flicking with(out) laser assistance) has not been standardized across studies, this issue has hardly been studied in relation to obstetric and neonatal outcomes. Operator dependency, but also intrinsic blastocyst quality to start from and relative TE cell number retrieved, represent unknown sources of heterogeneity and bias. In respect to different numbers of biopsied TE cells, one retrospective cohort study [99] has studied the impact on neonatal outcomes, showing a significantly different frequency in neonatal macrosomia (*p* = 0.037 in a very small subgroup of 51 singletons). Obviously, these data need to be confirmed (or contrasted) in larger sample sizes.

## 8. Conclusions and Future Perspectives

Different protocols for TE biopsy have emerged over time. Every change in the procedure should serve the goal to minimize manipulation and harm to the blastocyst potentially related to laser thermal heating. ZP opening by means of laser assistance represents the only standardized aspect of the procedure. The timing of zona breaching may differ between laboratories: day 3/day 4 versus day 5 opening, including immediate sequential TE cell removal. At present, one single prospective RCT showed significantly more blastocysts available for biopsy with the latter approach; however, this was not translated in a clinical benefit. In respect to TE cell removal, pulling can be considered as the standard procedure; given that blastocysts are not fully hatched, induced full hatching by manipulation can be avoided and the number of laser pulses for detachment can be limited. As an alternative, TE cells can be removed by mechanical flicking, which is preferably complemented by a few laser pulses before flicking to ease the biopsy and to precisely control the target number of TE cells to be detached. Neither the number of laser pulses nor the method, pulling or flicking, has been associated with increased artefactual mosaicism. Laser-free flicking should be considered with caution, as it represents a more rough and less precise technique with respect to cell number retrieved. It needs to be confirmed that it does not induce more damage to the blastocyst and the sample. Additionally, its operational impact on fair- and poor-quality blastocysts has not been validated so far.

The TE cell number to be biopsied (seven to eight cells) should minimize diagnostic test failures without impacting on blastocyst competence. Minimal embryo biomass reduction can be safeguarded by avoiding premature sampling when still insufficient cell numbers are present in the trophectoderm of the blastocyst. The inclusion of poor-quality and delayed blastocysts for biopsy should be considered, although their reduced prognosis needs to be acknowledged in counselling patients correctly. When poor-quality blastocysts are biopsied, operators should be careful not to include in their sample peripheral non-participating cells nor apoptotic remnants present in the blastocoelic cavity, as these are more often aneuploid or with highly fragmented DNA, not representing the remaining blastocyst (or its ICM) and potentially impacting on the sample quality.

The use of ICSI as the fertilization method ensures monospermic fertilization and avoids parental contamination. Whereas the continued use of ICSI seems appropriate for PGT-M and PGT-SR, IVF can be used for PGT-A in the absence of a paternal infertility factor given that maternal contamination is well controlled by careful denudation of the oocytes. Visual microscopic inspection should largely avoid the presence of (a) sperm cell(s) in biopsied TE samples. Evidence exists that sperm cell DNA amplification depends on the lysis conditions of the amplification strategy used. In order to increase the pool of blastocysts for biopsy, 0 PN and 1 PN blastocysts need to be considered given that the diagnostic test allows distinguishing haploidy (or triploidy) from diploidy.

So far, obstetric and neonatal outcome post-TE biopsy is reassuring; however, the confounding effect of infertility status cannot be excluded, as most studies have mixed PGT indications. Endometrial preparation of the FET cycles may also impact the obstetrical/neonatal outcome post-PGT. Additionally, as the methodology of the biopsy procedure has not been standardized across studies, this issue has hardly been studied in relation to safety in terms of neonatal outcome. Whereas re-biopsy in cases of diagnostic test failure has its clinical value (be it at a lower efficiency), the safety evaluation of double biopsy/double vitrification needs to be expanded with neonatal follow up.

## Abbreviations

ADO	allele drop out
ASRM	American Society for Reproductive Medicine
CPM	confined placental mosaicism
CVS	chorion villus sampling
DNA	deoxyribonucleic acid
ESHRE	European Society of Human Reproduction and Embryology
FET	frozen embryo transfer
HRT	hormone replacement therapy
ICM	inner cell mass
ICSI	intracytoplasmic sperm injection
IVF	in vitro fertilization
KPI	key performance indicator
LBR	live birth rate
MDA	multiple displacement amplification
MR	miscarriage rate
NC	natural cycle
NIPT	non-invasive prenatal testing
NGS	new generation sequencing
PCR	polymerase chain reaction
PGT	preimplantation genetic testing
PGT-A	preimplantation genetic aneuploidy testing
PGT-M	preimplantation genetic testing for monogenic disorders
PGT-SR	preimplantation genetic testing for structural rearrangements
PN	pronucleus
SET	single embryo transfer
SIG	special interest group
SNP	single nucleotide polymorphism
TE	trophectoderm
TL	time lapse
WGA	whole genome amplification
ZP	zona pellucida
